# Interleukin-4-Enhanced Oligodendrocyte Differentiation Depends on Extracellular Zinc Uptake via ZIP11

**DOI:** 10.3390/cells14221756

**Published:** 2025-11-10

**Authors:** Takaaki Aratake, Serika Kurita, Michael Wegner

**Affiliations:** Institut für Biochemie, Friedrich-Alexander-Universität Erlangen-Nürnberg, 91054 Erlangen, Germany; takaaki.aratake@fau.de (T.A.); serika.aratake@fau.de (S.K.)

**Keywords:** zinc, zinc transporter, ZIP11, IL-4, glia, oligodendrocyte

## Abstract

Differentiation of oligodendrocytes and myelination are enhanced by interleukin-4, an anti-inflammatory cytokine secreted from immune cells or injured neurons, and peroxisome proliferator-activated receptor γ serves as a central effector. While intracellular zinc concentrations have recently been reported to change dynamically during oligodendrocyte development, the role of zinc in interleukin-4-enhanced oligodendrocyte differentiation has not been studied. Using primary oligodendroglial cells and the oligodendroglial CG4 cell line, we show that intracellular zinc concentrations transiently increased 1 day after interleukin-4-induced differentiation and that intracellular as well as extracellular zinc chelators repressed the interleukin-4-dependent effects. Our analyses furthermore reveal that STAT6 activated the zinc transporter ZIP11 downstream of interleukin-4 in a phosphorylation-dependent manner and that siRNA-dependent knockdown of ZIP11 abolished the interleukin-4-enhanced oligodendrocyte differentiation. An antagonist of peroxisome proliferator-activated receptor γ similarly repressed the interleukin-4-dependent differentiation. However, agonists did not affect intracellular zinc concentrations. These findings indicate that interleukin-4 upregulates ZIP11 expression via activation of STAT6 and facilitates extracellular zinc uptake, which in turn activates peroxisome proliferator-activated receptor γ and thereby promotes oligodendrocytes differentiation. Our results argue that a modulation of zinc concentrations may be beneficial for promoting oligodendrocyte differentiation and remyelination under demyelinating conditions such as multiple sclerosis.

## 1. Introduction

Oligodendrocytes (OL) and astrocytes constitute the prevalent macroglial cell types of the central nervous system (CNS). The major roles of OL are to produce myelin sheaths, to speed up nerve conduction, and to provide metabolic support to neuronal axons. OL are generated from OL progenitor cells (OPC). The differentiation of OL from OPC requires the temporally controlled coordinate activity of several stage- and lineage-specific transcriptional regulators, among them several zinc-binding proteins such as Zeb2, Zfp24, Zfp276, and Zfp488 [1].

Zinc is the second most abundant trace element in the body following iron. In brain cells, approximately 70–80% of the total amount of zinc is bound to proteins, such as enzymes, signaling molecules, and transcription factors. As a consequence, the free cytosolic zinc concentration is considered to be very low under normal physiological conditions [2].

The disruption of zinc homeostasis is associated with several CNS disorders. In addition to stroke, Alzheimer’s disease, Parkinson’s disease, depression, schizophrenia, epilepsy, and traumatic brain injury, this also includes multiple sclerosis (MS), in which OL and their myelin sheaths are affected. Lower zinc levels were, for instance, observed in MS lesions compared to normal-appearing white matter [3]. Several studies have also suggested a lower serum zinc concentration in MS patients [4,5,6,7].

Intracellular zinc homeostasis is mainly maintained by zinc transporters. There are two types of zinc transporters, ZIP and ZnT proteins. ZIP proteins are zinc importers, and ZnT proteins are zinc exporters. In various cells, differentiation has been associated with intracellular zinc dynamics [8]. Recent studies have determined the zinc levels in OPC and OL and have reported increased intracellular zinc dynamics during OL differentiation [9,10]. Additionally, mutations in ZnT11 (known as TMEM163) cause CNS hypomyelination [11,12]. While this suggests that zinc may be involved in OL differentiation, its role has not been experimentally studied so far.

One of the signaling molecules that promotes OL differentiation is interleukin-4 (IL-4). IL-4 is an anti-inflammatory cytokine secreted by various immune cells throughout the body and acts via the interleukin-4 receptor alpha (Il4ra) [13]. In the CNS, IL-4 is additionally produced by injured neurons in ischemic brains [14]. OPC express Il4ra and thus are IL-4 responsive [15].

Recent work has investigated IL-4 as a therapeutic target for neurodegenerative diseases with a focus on immune cells such as microglia and peripheral immune cells. In contrast, there are only few reports about the direct effects of IL-4 on oligodendroglia. Those studies demonstrated that IL-4 promotes OL differentiation and remyelination in stroke and traumatic brain injury through the activation of peroxisome proliferator-activated receptor gamma (PPARγ), a zinc-containing member of the nuclear receptor superfamily [16,17,18].

Another well-known downstream effector of IL-4 signaling is the Signal transducer and activator of transcription 6 (STAT6). Higher expression of STAT6 has been observed in normal-appearing white matter of MS patients compared to healthy controls [19,20]. It was demonstrated that STAT6 protein is expressed mainly in OL [19]. STAT6 expression was increased even further in the lesion site compared to normal-appearing white matter [21]. These data suggest that the IL-4/STAT6 signaling pathway plays a key role in OL differentiation and (re)myelination under pathophysiological conditions.

Taking into account that IL-4 increases the cytosolic zinc level in microglia and macrophages [22,23], we postulated that increased intracellular zinc concentrations are also involved in the IL-4 effects on OL differentiation. Indeed, the current study revealed for the first time that IL-4 promotes OL differentiation via zinc by a mechanism that involves increased extracellular zinc uptake by ZIP11 downstream of STAT6 and upstream of PPARγ.

## 2. Materials and Methods

All chemicals and reagents were obtained from Sigma-Aldrich (Hamburg, Germany) unless otherwise noted.

### 2.1. Cell Culture and Transfection

Primary oligodendroglial cultures were prepared from mixed glial cultures obtained from newborn Wister rats, as previously described [24]. For propagation, primary oligodendroglial cells were seeded on poly-ornithine (P3655) coated cell culture dishes and maintained in basal medium supplemented with 10 ng/mL PDGF-AA (PeproTech, Rocky Hill, NJ, USA) and 10 ng/mL FGF2 (PeproTech).

Rat oligodendroglial CG4 cells [25] were maintained in basal medium supplemented with 10 ng/mL PDGF-AA and 10 ng/mL FGF2, as described previously [26]. They were transfected with 25 nM siRNA pools for Slc39a11 (Dharmacon, #M-086493-01, Lafayette, CO,) or siGENOME non-targeting siRNA #1 (Dharmacon, #D-001210-01), using Lipofectamine 2000 (Thermo Fisher Scientific, Dreieich, Germany) for 24 h under proliferative conditions.

For the experiments reported in this study involving immunocytochemistry and flow cytometry, primary oligodendroglial and CG4 cells were seeded on 12-well plates at a density of 10^5^ cells per poly-ornithine-coated well in the above-mentioned proliferation medium for at least a day before the start of the experiment. In the case of immunocytochemical analysis, the well contained a poly-ornithine-coated coverslip. For experiments using qRT-PCR or Western blot, poly-ornithine-coated 6 cm dishes were used at a density of 10^6^ cells. Differentiation was induced by replacing the basal medium with a serum-free SATO medium containing T3/T4. Half the differentiation medium was replaced every other day. The following substances were added during differentiation alone or in various combinations: IL-4 (20 ng/mL, PeproTech), TPEN (1 μM), CaEDTA (100 μM), the STAT6 inhibitor AS1517499 (10, 100, or 300 nM), the PPARγ agonist pioglitazone (1 μM), and the PPARγ antagonist GW9662 (1 μM).

### 2.2. Immunocytochemistry

Cells were fixed with 4% paraformaldehyde. After blocking with 1% BSA and 10% FCS (Anprotec, Bruckberg, Germany, #AC-SM-0143) in PBS, the cells were permeabilized with PBS containing 0.1% triton-X100 and then incubated with primary and secondary antibodies. The following primary antibodies were used: rat anti-Mbp (1:750, #MCA409S, Bio-Rad, Eschborn, Germany), rabbit anti-Plp1 (1:1,000, #ab28486, Abcam, Cambridge, MA, USA), goat anti-Sox10 (1:5000, made in-house) [27], mouse anti-Olig2 (1:100, #MABN50, Millipore, Burlington, MA, USA), mouse anti-O4 (1:500, #MAB1326, R&D Systems, Minneapolis, MN, USA), rabbit anti-pSTAT6 (1:200, #56554S, Cell Signaling, Beverly, MA, USA), and rabbit anti-ZIP11 (1:400, #PA5-20679, Invitrogen, Waltham, MA, USA). Secondary antibodies were conjugated to Alexa Fluor 488 (1:500, Molecular Probes, Eugene, OR, USA), Alexa Fluor 546 (1:500, #A-21123, Invitrogen), Cy3 or Cy5 (1:200, Dianova, Hamburg, Germany) fluorescent dyes. Nuclei were counterstained with 4,6-Diamidine-2-phenylindole (DAPI). The images were documented on a Leica DMI 6000B inverted microscope (Leica, Wetzlar, Germany) or Zeiss ApoTome 2 (Carl Zeiss Microscopy GmbH, Jena, Germany) and analyzed using ImageJ (https://imagej.net/ij/). If not otherwise stated in the figure legends, Leica microscope images are shown. Sox10 is a nuclear marker colocalizing with DAPI in oligodendroglial cells; the two myelin proteins Mbp and Plp1 also colocalize (Supplementary Figure S1).

For quantifications of immunocytochemical staining, we photographed three fields per coverslip and used three coverslips in each sample of each experiment. To quantify the ZIP11 levels, we measured the mean fluorescence intensity of whole-cell ZIP11 signals. To quantify pSTAT6 levels in nuclei, we segmented 1500–2000 nuclei per condition and specimen using celldetection (v0.4.5) [28] and the pretrained ginoro_CpnResNeXt101UNet-fbe875f1a3e5ce2c model. For nucleus segmentation, we utilized Olig2 and DAPI staining images in primary oligodendroglial and CG4 cells, respectively. The protein levels of pSTAT6 in each segmented nucleus were quantified by employing measure.regionprops of the scikit-image package (v0.21.0) in Python (v3.8.18). The integrated intensity (sum of the intensity of all pixels in a segmented nucleus) was used for statistical analysis.

### 2.3. Intracellular Zinc Concentration Measuring by Flow Cytometry

Primary rat oligodendroglial cells were loaded with 1 μM FluoZin-3 AM (Thermo Fisher Scientific) in culture medium at 37 °C for 30 min, washed with culture medium, and incubated with 100 μM *N*,*N*,*N*,*N*-tetrakis(2-pyridinylmethyl)-1,2-ethanediamine (TPEN) or 10 μM zinc pyrithione at 37 °C for 30 min. The cells were detached from the plates using accutase and collected in a 1.5 mL tube. To examine the cellular viability, the cells were incubated with LIVE/DEAD Fixable Near-IR Dead Cell Stain Kit for 633 or 635 nm excitation (Invitrogen) for 30 min at 4 °C in the dark. Data were acquired on a FACS-LSR Fortessa (Becton Dickinson, Franklin Lakes, NJ, USA) and were analyzed with FlowJo v10.6.0 (Becton Dickinson). Zinc concentrations were calculated essentially, as previously described [29].

### 2.4. Quantitative RT-PCR (qRT-PCR)

The total RNA was extracted from primary oligodendroglial and CG4 cells using Trizol (Invitrogen). After reverse transcription using an oligo dT primer, qRT-PCR was performed on the Biorad CFX96 Real Time PCR System (Bio-Rad) using PowerUp SYBR Green Mastermix (Thermo Fischer Scientific, Waltham, MA, USA). PCR amplification was performed using the gene specific primer sets shown in Table 1. The target gene mRNA expression was normalized to glyceraldehyde 3-phosphate dehydrogenase (*Gapdh*) mRNA expression, and the relative amounts of all mRNAs were calculated using the comparative Ct method.

### 2.5. Western Blotting

Whole cell lysates were produced from CG4 cells. Samples were run on 10% polyacrylamide sodium-dodecyl-sulfate gels and proteins consecutively transferred to nitrocellulose membranes. After blocking with 5% skim milk in Tris-buffered saline containing Tween-20 for 1 h, membranes were incubated with the following primary antibodies: rabbit anti-ZIP11 (1:1000, #PA5-20679, Invitrogen) and rabbit anti-GAPDH (1:1000, #SC-25778, SantaCruz, CA, USA). Protein A-HRP conjugates (1:3000 dilution, Zymed, Vienna, Austria) were used as a secondary antibody. Signals were detected using a SuperSignal West Femt Maximum Sensitivity Substrate (Thermo Scientific) and visualized using the Azure 300 (Azure Biosystems, Munich, Germany). Band intensities were quantified using the ImageJ plugin Fiji.

### 2.6. Statistical Analysis

All data are shown as the mean ± standard deviation (SD). All experiments were performed with *n* = 4, if not otherwise stated in the figure legends. To determine whether the differences were statistically significant between two or more groups, an unpaired two-tailed Student’s *t* test or one-way or two-way ANOVA with Tukey’s multiple comparison test was performed using GraphPad Prism 8 (GraphPad software, San Diego, CA, USA). Differences with a *p*-value of 0.05 or less were considered statistically significant.

## 3. Results

### 3.1. IL-4 Increases Transiently Intracellular Zinc Concentrations in Differentiating Oligodendroglia

**Figure 1:**
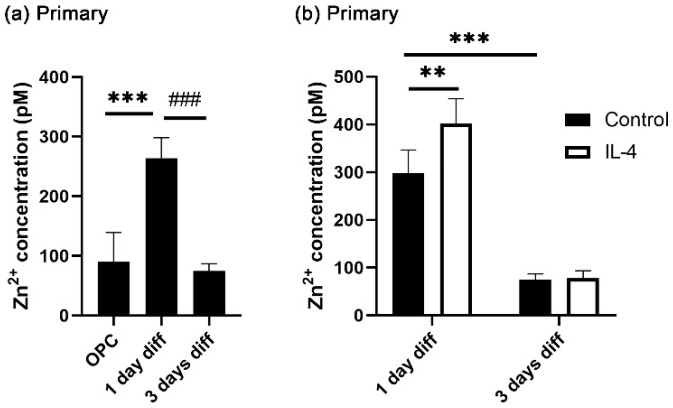
Intracellular zinc dynamics in oligodendroglia. After 1 day in culture, primary oligodendroglial cells were differentiated for 1 or 3 days in the presence of 20 ng/mL IL-4. (**a**,**b**) Quantification of intracellular zinc concentrations in various oligodendroglial differentiation states in the presence of IL-4 by flow cytometry, shown as mean ± SD (*n* = 4). Experiments in (**a**,**b**) represent separate trials. Statistical significance was determined by one-way ANOVA with Tukey’s multiple comparison test for (**a**) and by two-way ANOVA with Tukey’s multiple comparison test for (**b**) (**, *p* ≤ 0.01; *** and ^###^, *p* ≤ 0.001).

Recent studies reported that intracellular zinc concentrations are transiently increased during OL differentiation [9,10]. To confirm this, cytosolic zinc concentrations were measured in cultures of primary rat oligodendroglial cells during the course of differentiation using flow cytometry. Differentiation was induced by switching the culture medium from proliferative (low T3 concentrations in the presence of FGF2 and PDGF-AA) to differentiation-promoting (high T3 concentrations in the absence of FGF2 and PDGF-AA) conditions. In line with the published reports, we detected higher zinc concentrations in cells kept for one day in differentiating conditions than in cells before the onset of differentiation (Figure 1a). By three days, the zinc concentrations had returned to the levels found in proliferative conditions (Figure 1a). As shown in Figure 1b, in the presence of IL-4, intracellular zinc concentrations were increased even higher at one day of differentiation, indicating that the reported differentiation-promoting activity of IL-4 in OL [16,17] may be linked to its impact on intracellular zinc concentrations. At 3 days after differentiation, the effect of IL-4 on cytosolic zinc concentrations was no longer detected.

### 3.2. Increased OL Differentiation by IL-4 Is Zinc-Dependent

**Figure 2:**
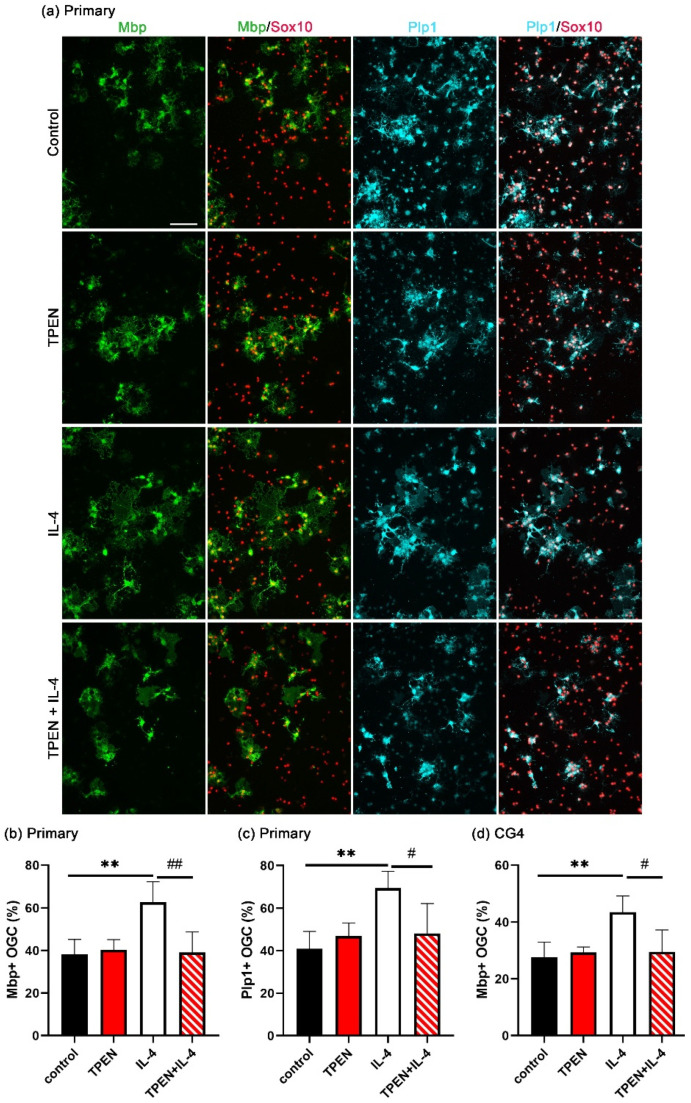
The effect of TPEN on IL-4-dependent OL differentiation. After 1 day in culture, differentiation was induced in the presence of TPEN (1 µM) and/or IL-4 (20 ng/mL) for 3 days. (**a**) Immunocytochemical visualization of mature OL markers (Mbp and Plp1) and a pan-oligodendroglial marker (Sox10). Scale bar = 100 μm. (**b**,**c**) Quantification of the fraction of Mbp- (**b**) or Plp1- (**c**) positive Sox10-labeled oligodendroglial cells (OGC) as mean ± SD (*n* = 4). (**d**) Quantification of the fraction of Mbp-positive Sox10-labeled CG4 cells as mean ± SD (*n* = 4). The density of Sox10-positive cells varied not more than 25% for OGC and 13% for CG4 cells between samples without bias for specific conditions. Statistical significance was determined by one-way ANOVA with Tukey’s multiple comparison test (^#^, *p* ≤ 0.05; ** and ^##^, *p* ≤ 0.01).

To investigate whether increased intracellular zinc is essential for IL-4-induced OL differentiation, the zinc chelator TPEN was added to cultures of differentiating OL, and the differentiation was assessed by immunohistochemistry for the myelin proteins Mbp or Plp1. After three days of differentiation, around 40% of the Sox10-positive oligodendroglial cells had become positive for Mbp under control conditions (Figure 2b). This percentage did not change when the cells were treated with TPEN alone.

As previously demonstrated, the addition of IL-4 significantly enhanced the percentage of Mbp-positive cells after three days of differentiation compared to standard conditions. However, this enhancement was suppressed by TPEN. As shown in Figure 2c, similar results were obtained when the rate of differentiation was monitored by Plp1 as a second marker for myelinating OL. These results indicated that increases in intracellular zinc concentrations are not essential for T3-driven OL differentiation but are required for the differentiation-promoting effects of IL-4.

We also performed these experiments in rat CG4 cells. The CG4 cell line is derived from immortalized rat oligodendroglia [25]. It represents a good model system to study oligodendroglial cells, because CG4 cells are able to turn on the oligodendroglial differentiation program in culture and have a very similar transcriptomic profile to primary oligodendroglial cells under both proliferative and differentiating conditions [30]. Compared to primary oligodendroglial cells, they differentiate less well. However, they have an advantage in that they do not contain low numbers of contaminating microglia and are much more amenable to experimental manipulation. Therefore, it was reassuring that IL-4-treatment also enhanced the number of Mbp-positive CG4 cells after three days of differentiation compared to the control conditions, and this enhancement was again inhibited by TPEN (Figure 2d).

### 3.3. IL-4 Induces Extracellular Zinc Uptake

**Figure 3:**
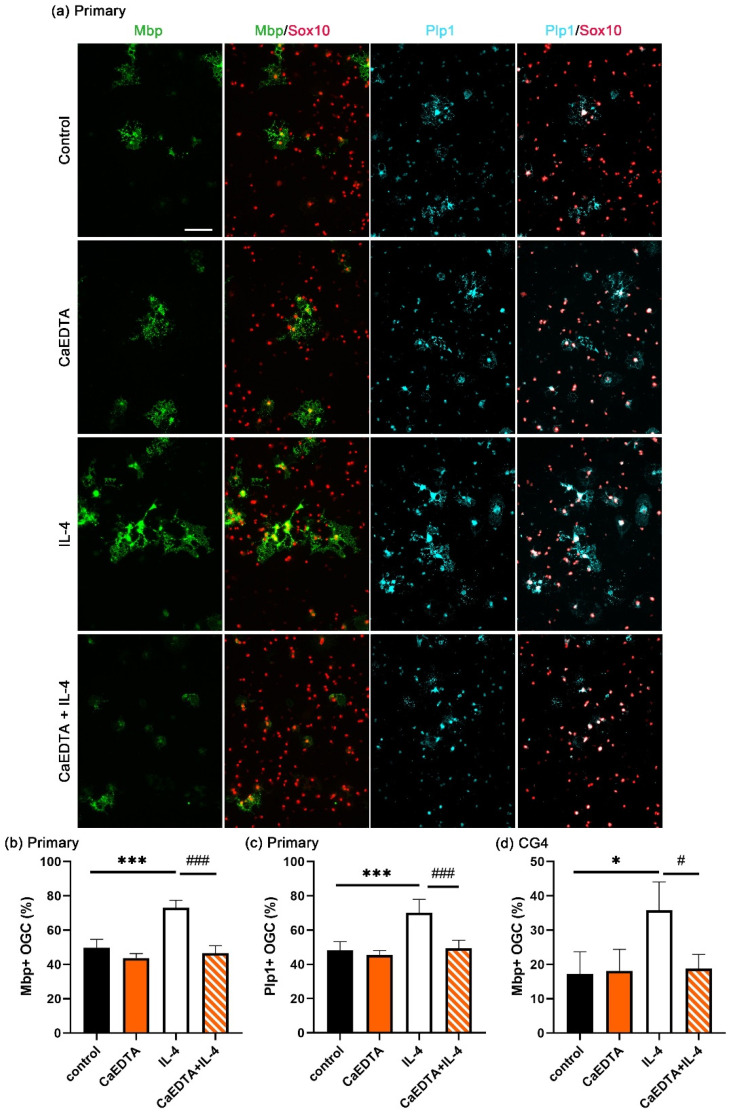
The effect of CaEDTA on IL-4-enhanced OL differentiation. Differentiation was induced in the presence of CaEDTA (100 µM) and/or IL-4 (20 ng/mL) for 3 days. (**a**) Immunocytochemical visualization of mature OL markers (Mbp and Plp1) and a pan-oligodendroglial marker (Sox10). Scale bar = 100 μm. (**b**,**c**) Quantification of the fraction of Mbp- (**b**) or Plp1- (**c**) positive Sox10-labeled primary rat oligodendroglial cells (OGC) as mean ± SD (*n* = 4). (**d**) Quantification of the fraction of Mbp-positive Sox10-labeled CG4 cells as mean ± SD (*n* = 3). The density of Sox10-positive cells varied not more than 35% for OGC and 14% for CG4 cells between samples without bias for specific conditions. Statistical significance was determined by one-way ANOVA with Tukey’s multiple comparison test (* and ^#^, *p* ≤ 0.05; *** and ^###^, *p* ≤ 0.001).

TPEN is a membrane permeable zinc chelator, and TPEN can chelate both intracellular and extracellular zinc ions. To determine the origin of the zinc that is required for the enhancement of OL differentiation by IL-4, differentiation was studied in the presence of the non-membrane permeable zinc chelator CaEDTA instead of TPEN. Under these conditions, only the extracellular zinc was chelated. CaEDTA behaved very similar to TPEN in primary oligodendroglial cultures, as it had little effect on the percentage of Mbp- and Plp1-positive OL generated during a three-day differentiation period in the absence of IL-4 but completely suppressed the IL-4-dependent enhancement of OL differentiation (Figure 3a–c). CaEDTA similarly prevented IL-4 from increasing CG4 cell differentiation (Figure 3d). The zinc that is required for the positive effects of IL-4 on OL differentiation must therefore be of an extracellular origin.

### 3.4. STAT6 Activation Is Essential for Extracellular Zinc Uptake

**Figure 4:**
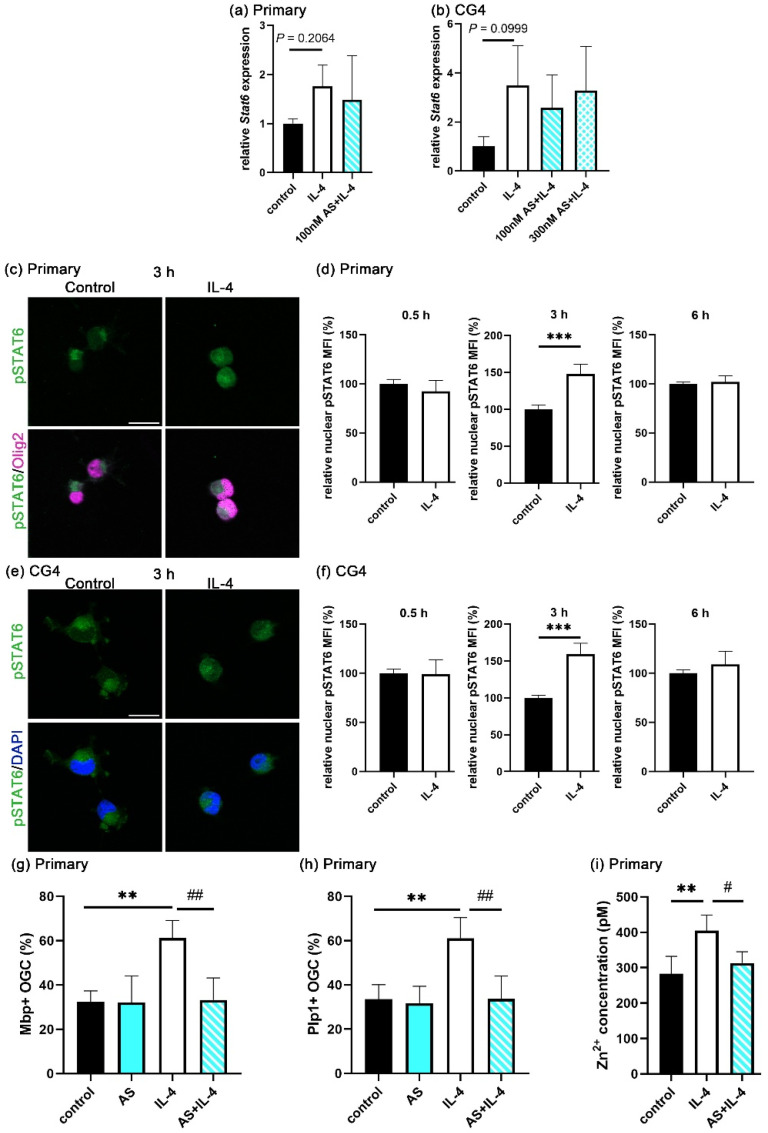
STAT6 activation in oligodendroglia. (**a**,**b**) *Stat6* transcript levels in primary rat oligodendroglia (**a**) and in CG4 cells (**b**) treated with IL-4 (20 ng/mL) for 24 h and, where indicated, AS (100 and 300 nM), according to qRT-PCR. The mRNA levels of *Stat6* were normalized to the *Gapdh* levels and are presented as mean ± SD (*n* = 4). (**c**–**f**) Immunocytochemical visualization of pSTAT6 in single plane captured by Apotome (**c**,**e**) and merged with Olig2 in primary cells (**c**) or DAPI in CG4 cells (**e**) treated with IL-4 (20 ng/mL) for 3 h. Scale bar = 20 μm. From this and comparable staining at 0.5 and 6 h, nuclear pSTAT6 levels were determined after nuclear segmentation and are presented as mean fluorescence intensity (MFI) ± SD in primary oligodendroglial (**d**) and CG4 (**f**) cells (*n* = 4). (**g**,**h**) Quantification of the fraction of Mbp- (**g**) and Plp1- (**h**) positive Sox10-labeled oligodendroglial cells (OGC) after 3 days of differentiation in the presence of IL-4 (20 ng/mL) ± AS (100 nM) and/or IL-4, *n* = 4). The density of Sox10-positive cells varied not more than 28% between samples without bias for specific conditions. (**i**) Determination of intracellular zinc concentrations in primary cells after 1 day in the presence of IL-4 (20 ng/mL) ± AS (100 nM). Data are shown as mean ± SD (*n* = 4). Statistical significance was determined by two-tailed Student’s *t*-test for (**b**,**d**,**e**) or one-way ANOVA with Tukey’s multiple comparison test for (**a**, **g**–**i**) (^#^, *p* ≤ 0.05; ** and ^##^, *p* ≤ 0.01; ***, *p* ≤ 0.001).

STAT6 is a main downstream effector of IL-4 signaling. To assess the role of STAT6, we first analyzed its expression during the course of IL-4-enhanced OL differentiation in both primary oligodendroglial cells and CG4 cells. By qRT-PCR, we detected a mild increase in *Stat6* mRNA levels in the IL-4-treated cells as compared to the controls. However, this increase was not statistically significant (Figure 4a). When similar experiments were performed on the oligodendroglial CG4 cell line, we also observed higher *Stat6* levels, again without statistical significance (Figure 4b).

Upon IL-4 binding to its receptor, STAT6 iss phosphorylated and relocalizes to the nucleus. Therefore, the amount of phospho-STAT6 (pSTAT6) in the nucleus is the decisive determinant for the IL-4 response. At 30 min of differentiation, pSTAT6 was not yet observed in nuclei at substantial levels. In contrast, the nuclear translocation of pSTAT6 occurred at 3 h after differentiation in significant amounts (Figure 4c,e). When we measured the signal intensity for nuclear pSTAT6 after segmentation of 1500–2000 nuclei per condition and sample, we detected a significantly higher intensity in the IL-4-treated group as compared to the controls at 3 h of differentiation, but not at 30 min and 6 h of differentiation (Figure 4d,f). The transient increase in nuclear pSTAT6 was observed both in primary OL and CG4 cells. To analyze whether pSTAT6 contributes to the IL-4-dependent OL differentiation, we applied the STAT6 phosphorylation inhibitor AS1517499 (AS) to differentiating cultures. We first investigated the effect of AS on *Stat6* mRNA levels by qRT-PCR. As expected, AS did not affect the IL-4-induced *Stat6* mRNA levels in primary oligodendroglial cells or CG4 cells (Figure 4a,b). With regard to OL differentiation, AS acted similar to zinc chelators and completely inhibited the IL-4-dependent increase in Mbp- and Plp1-positive cells after three days of differentiation (Figure 4g,h).

Since pSTAT6 levels transiently increased during IL-4-enhanced OL differentiation, we also investigated its effect on the intracellular zinc concentration. For that purpose, we measured the intracellular zinc concentrations in differentiating OL at one day of differentiation in the presence of AS. As shown in Figure 4i, AS reduced the IL-4-dependent increase in intracellular zinc concentrations to the levels observed in the absence of IL-4. We conclude from these data that AS inhibits the IL-4-induced extracellular zinc uptake, arguing that STAT6 activation is essential for the transient increase in the extracellular zinc influx and subsequent IL-4-dependent OL differentiation.

### 3.5. Zinc Influx via ZIP11 Increases Intracellular Zinc

One way, in which STAT6 could increase the influx of extracellular zinc is by stimulating the expression of relevant zinc transporters. To predict relevant zinc transporter genes, we utilized the Harmonizome 3.0 database [31] and found *Slc39a8* and *Slc39a11* as potential target genes of STAT6.

**Figure 5:**
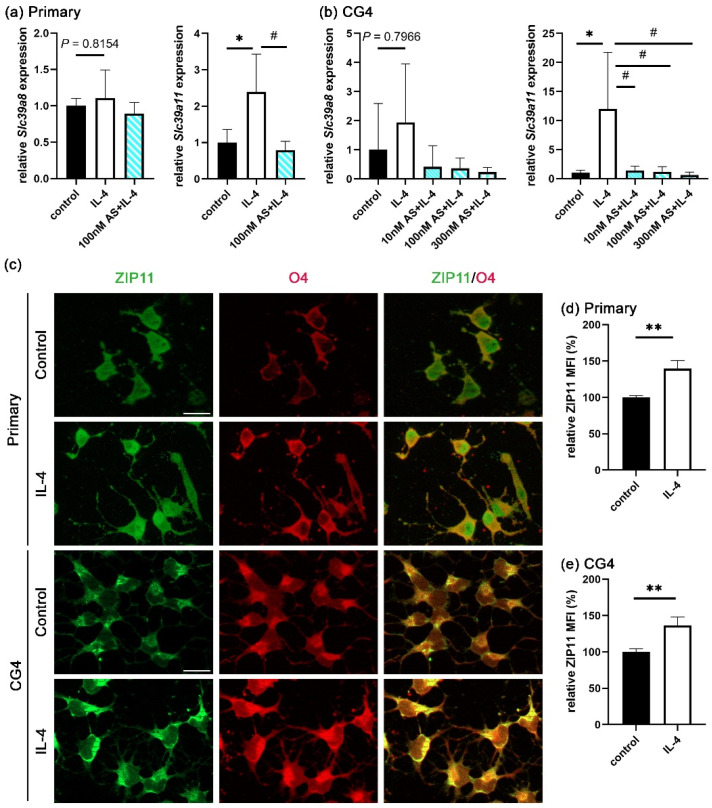
ZIP transporter expression profiles and localization in oligodendroglia. (**a**,**b**) Zinc transporter transcript levels in primary oligodendroglial cells (**a**) and CG4 cells (**b**) treated with IL-4 (20 ng/mL) in the absence or presence of AS (10, 100, and 300 nM) for 24 h, as determined by qRT-PCR. The mRNA levels were normalized to the *Gapdh* levels and are presented as the mean ± SD (*n* = 4). (**c**) Immunocytochemical visualization of Zip11 in single plane captured by Apotome and merged with O4 in primary cells or CG4 cells treated for 1 day with IL-4 (20 ng/mL). Scale bar = 20 μm. (**d**,**e**) Quantification of ZIP11 mean fluorescence intensity ± SD (*n* = 3) in primary (**d**) and CG4 (**e**) cells kept 1 day in the absence or presence of IL-4 (20 ng/mL). Statistical significance was determined by one-way ANOVA with Tukey’s multiple comparison test for (**a**,**b**) or two-tailed Student’s *t*-test for (**d**,**e**) (* and ^#^, *p* ≤ 0.05; **, *p* ≤ 0.01).

As shown by qRT-PCR (Figure 5a,b), *Slc39a8* transcripts appeared slightly increased in both the IL-4-treated primary oligodendroglial and CG4 cells compared to the controls. However, the increase did not reach statistical significance. On the other hand, *Slc39a11* mRNA levels were significantly upregulated by IL-4 treatment. Intriguingly, the STAT6 inhibitor AS completely prevented the IL-4-induced *Slc39a11* mRNA expression in both primary oligodendroglial and CG4 cells but did not affect the *Slc39a8* mRNA levels. In addition, published RNAseq data indicated much higher expression levels for *Slc39a11* than for *Slc39a8* in oligodendroglial cells [15]; hence, we focused on *Slc39a11*.

**Figure 6:**
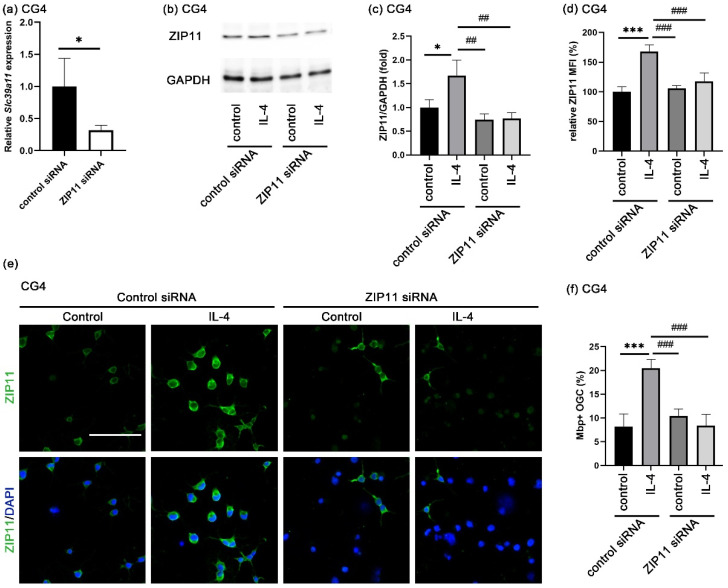
Consequences of ZIP11 loss in IL-4-enhanced OL differentiation. (**a**) Determination of ZIP11 mRNA levels (*Slc39a11*) in CG4 cells incubated with control and ZIP11-specific siRNA (25 nM) for 24 h by qRT-PCR. Values were normalized to *Gapdh* mRNA levels and are presented as mean ± SD (*n* = 4). (**b**,**c**) Western blot analysis on whole cell lysates of CG4 cells first incubated for 1 day with siRNA (25 nM) and then treated for another day with IL-4 (20 ng/mL), with antibodies directed against ZIP11 and GAPDH (**b**) and quantification of band intensities (**c**). ZIP11 values were normalized to GAPDH and are presented as mean ± SD (*n* = 3). (**d**,**e**) Quantification of mean ZIP11 fluorescence intensity ± SD in CG4 cells (**d**, *n* = 4) according to immunocytochemical staining with antibodies directed against ZIP11, after consecutive treatment with siRNA (25 nM) for 24 h and IL-4 (20 ng/mL) for 24 h (**e**). DAPI was used to visualize the nuclei. Scale bar = 100 μm. (**f**) Quantification of the fraction of Mbp-positive Sox10-labeled CG4 after 24 h incubation with siRNA (25 nM) and 3 additional days of differentiation in the presence or absence of IL-4 (20 ng/mL) according to immunocytochemical staining (*n* = 4). Statistical significance was determined by two-tailed Student’s *t*-test (**a**) or by two-way ANOVA with Tukey’s multiple comparison test (**c**,**d**,**f**) (*, *p* ≤ 0.05; ^##^, *p* ≤ 0.01; *** and ^###^, *p* ≤ 0.001).

ZIP11, the zinc transporter encoded by the *Slc39a11* gene, has been reported to be localized in the nuclear membrane and the membrane of the Golgi apparatus [32,33,34]. Although ZIP11 has been considered as an extracellular zinc importer, there is no staining evidence that ZIP11 is localized in the plasma membrane [35]. Therefore, we investigated by immunocytochemistry where ZIP11 proteins are localized in oligodendroglial cells. As is evident from Figure 5c, specific staining for ZIP11 was found in cultures of both primary oligodendroglial and CG4 cells. ZIP11 was not only found in the cytoplasm and nucleus, but also in the plasma membrane, where it colocalized with the OL-specific epitope recognized by the O4 antibody. IL-4 caused an increase in the total cellular ZIP11 fluorescence intensity at 1 day after IL-4 treatment in both primary oligodendroglial and CG4 cells compared to the control (Figure 5d,e and Figure 6b,c). This suggests that STAT6 activation is responsible for the upregulation of ZIP11 expression.

To study whether ZIP11 could be involved in the IL-4-dependent increase in extracellular zinc uptake, we examined the effect of the ZIP11 loss on the IL-4-enhanced OL differentiation. In CG4 cells, *Slc39a11* mRNA was successfully reduced by transfection with ZIP11-specific siRNA (Figure 6a). We also confirmed an efficient knockdown on the protein level. ZIP11 protein was lower after transfection with ZIP11-specific siRNA than after transfection with a control siRNA according to Western blots (Figure 6b,c) and immunocytochemical staining (Figure 6d,e). When CG4 cells were differentiated for three days in the presence of control or ZIP11-specific siRNA, only the cells transfected with control siRNA were still able to respond to IL-4 with enhanced differentiation (Figure 6f). ZIP11 knockdown prevented the IL-4-dependent enhancement (Figure 6f). These data suggest that ZIP11 is involved in the extracellular zinc uptake under IL-4 treatment.

### 3.6. IL-4-Induced Intracellular Zinc Accumulation Promotes the PPARγ Signaling Pathway

**Figure 7:**
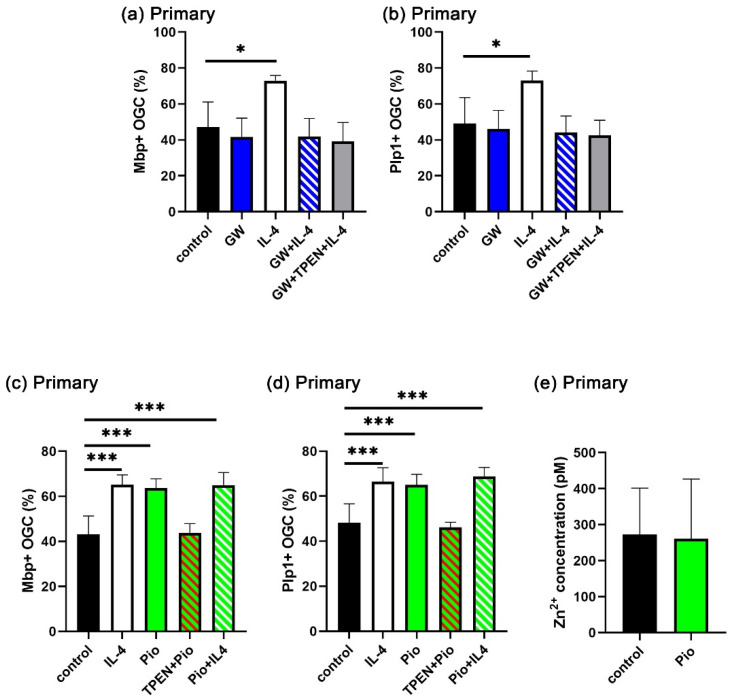
Involvement of PPARγ in IL-4-dependent OL differentiation. (**a**–**d**) Quantification of the fraction of Mbp- (**a**,**c**) or Plp1-positive (**b**,**d**) Sox10-labeled primary oligodendroglial cells (OGC) after 3 days of differentiation in the presence of GW9662 (GW, 1 µM), pioglitazone (Pio, 1 µM), TPEN (1 µM), and IL-4 (20 ng/mL), or various combinations thereof, as mean ± SD (*n* = 4). The density of Sox10-positive cells varied not more than 20% (**a**,**b**) and 22% (**c**,**d**) between samples without bias for specific conditions. (**e**) Intracellular zinc concentration in primary cells after incubation with pioglitazone (1 µM) for 24 h as mean ± SD (*n* = 4). Statistical significance was determined by one-way ANOVA with Tukey’s multiple comparison test for (**a**–**d**) or two-tailed Student’s *t*-test for (**e**) (*, *p* ≤ 0.05; ***, *p* ≤ 0.001). For representative images, see Supplementary Figure S2.

Our next experiments focused on the question of whether increased intracellular zinc levels play a role in the IL-4/STAT6 signaling pathway. PPARγ is a downstream effector of IL-4/STAT6 signaling. As a member of the family of nuclear receptors, it contains two zinc fingers and may thus be sensitive to intracellular zinc concentrations. Therefore, we investigated whether PPARγ is involved in the IL-4-dependent enhancement of OL differentiation and whether it is sensitive to the intracellular zinc concentration in that function. First, we used the PPARγ antagonist GW9662 (GW) and combined it in OL differentiation assays with IL-4 and TPEN (Figure 7a,b, Supplementary Figure S2). GW did not affect the rate of oligodendroglial cells converting to an Mbp- and Plp1-positive state during three days in high T3 medium, but it completely suppressed the IL-4-dependent enhancement. The additional presence of TPEN had no further effect. This result indicates that IL-4 requires PPARγ to enhance the rate of OL differentiation.

When the PPARγ agonist pioglitazone (Pio) was added to the cultures of differentiating OL instead of IL-4, we observed a similar increase in the rate of Mbp- and Plp1-positive cells over three days, as with IL-4 (Figure 7c,d, Supplementary Figure S2). Importantly, TPEN prevented the Pio-dependent increase in differentiating OL, just as it prevented the effects of IL-4. There was no additive effect on the rate of OL differentiation in the joint presence of IL-4 and Pio. However, in contrast to IL-4, the presence of Pio did not lead to a transient increase in intracellular zinc concentrations at one day of differentiation (Figure 7e). These data indicate that IL-4 increased cytosolic zinc concentrations and PPARγ act in the same pathway to enhance OL differentiation. Furthermore, it appears likely that the IL-4-induced intracellular zinc increase is upstream of PPARγ and acts at least in part via PPARγ signaling.

## 4. Discussion

Although many researchers have pointed out that zinc plays a critical role in remyelination in MS [36,37], a detailed mechanism has not been described so far. By outlining the effects of zinc during IL-4-dependent OL differentiation and comparing them with those of other IL-4 effectors, we provide mechanistic insights into the action of zinc in oligodendroglia.

In the literature, the standard intracellular zinc concentration is reported to be in the range of tens to hundreds pM [38]. Using two-photon microscopy with a ratiometric fluorescent zinc probe, zinc concentrations were, for instance, determined around 135–152 pM in developing OL and around 65–95 pM in mature OL [10]. Furthermore, intracellular zinc concentrations have been reported to increase transiently during differentiation [39,40,41,42]. In oligodendroglial cells, a study reported lower intracellular zinc levels in OPC and mature OL than in oligodendroglial cells immediately after induction of differentiation [9]. This agrees well with the concentrations determined in our study. We found zinc concentrations around 80 pM in the OPC state, approximately 300 pM one day after the onset of differentiation and 80 pM again 3 days into the differentiation process. The addition of IL-4 further increased the intracellular zinc concentrations to approximately 400 pM one day of differentiation. It has previously been shown that the rise in intracellular zinc concentrations is followed by an upregulation of metallothioneins and the zinc exporter ZnT1 as components of the intracellular zinc maintenance system in differentiating OL [9]. At least in macrophages, IL-4 has also been reported to induce metallothioneins and zinc exporters (i.e., ZnT4) [23]. Therefore, it seems reasonable to assume that an IL-4-dependent upregulation of metallothioneins and zinc exporters, after the rise in free cytosolic zinc concentrations, is likely responsible for the transient nature of the increase and the return to baseline levels at three days after differentiation.

Zinc is a critical structural component in myelin-related proteins such as Mbp and Mag, which in turn are required for the compaction of myelin [43,44,45]. Therefore, the transient increase in intracellular zinc levels in differentiating OL was expected. The surprising finding was that we did not observe an effect of TPEN under standard differentiating conditions. However, this does not mean that zinc has no effect on standard OL differentiation. For one, we only used the induction of the myelin components Mbp and Plp1 as a simplified readout for differentiation. Additionally, we only studied the differentiation after a 3-day period. Therefore, we cannot rule out that other parameters of OL differentiation would be affected more dramatically or that an analysis after 6 days may have been better suited for detecting changes in Mbp and Plp1. It is, for instance, utterly plausible that intracellular zinc stores (e.g., zinc bound to metallothioneins) are sufficient to carry OL differentiation through the first days and that depletion of zinc stores becomes visible only later or under conditions where OL differentiation is further enhanced, for instance by IL-4 treatment.

IL-4 has previously been reported to increase intracellular zinc levels in microglia and macrophages [22,23]. In these cells, zinc was largely mobilized from intracellular zinc stores. Therefore, it was surprising that the increase in oligodendroglial cells was primarily by uptake of extracellular zinc and, thus, by a pathway distinct from that in immune cells.

STAT6 is one of the well-known downstream effectors of IL-4 signaling. Upon IL-4 binding to its receptor, STAT6 is phosphorylated by JAK, translocates to the nucleus, and then acts as a transcription factor. Many studies have reported STAT6 phosphorylation 0.5–24 h after stimulation. We observed higher pSTAT6 expression levels in the nucleus at 3 h of IL-4 treatment, but we no longer detected a difference between the control and IL-4 treated cells at 6 h. Importantly, a STAT6 phosphorylation inhibitor prevented the intracellular zinc increase, arguing that STAT6 is upstream.

PPARγ is another major effector of IL-4 signaling. Several studies have demonstrated that OL differentiation is promoted by PPARγ agonists [46,47,48,49]. Furthermore, IL-4 treatment failed to enhance oligodendrogenesis and differentiation in oligodendroglial PPARγ conditional knockout mice after ischemic and traumatic brain injury [16,17]. In line with these data, we found that the PPARγ agonist pioglitazone increased OL differentiation as efficiently as IL-4, whereas the PPARγ antagonist GW9662 interfered with IL-4-dependent OL differentiation. Unexpectedly, however, pioglitazone did not increase the intracellular zinc concentration despite promoting OL differentiation. Therefore, PPARγ is downstream of zinc. Considering that PPARγ has also been reported to be downstream of STAT6 [50,51], it seems reasonable to assume that PPARγ expression is induced by STAT6 and that the increase in zinc is additionally required for PPARγ activation. As a member of the nuclear receptor family, PPARγ requires zinc for DNA-binding and dimerization.

With the increase in intracellular zinc concentrations being primarily dependent on the uptake of extracellular zinc, we searched among ZIP proteins for potential zinc importers. The Harmonizome 3.0 database predicted both ZIP11 and ZIP8 to be modulated in their expression by STAT6 [31]. In this study, we demonstrated that IL-4 indeed increased ZIP11 but not ZIP8 expression in oligodendroglia. The increase in ZIP11 expression was furthermore prevented by the STAT6 phosphorylation inhibitor AS. Importantly, ZIP11 knockdown also prevented an IL-4-induced increase in OL differentiation. Therefore, ZIP11 is a good candidate for mediating the IL-4-induced zinc uptake.

So far, ZIP11 has been documented in the nuclear membrane and the membrane of the Golgi apparatus in several cell types [32,33,34,52]. Although presumed to be localized in the plasma membrane as well [35], our results present the first experimental evidence for a ZIP11 localization in the plasma membrane. A recent study also reported that ZIP11 can act as a manganese transporter [53]. However, the two zinc chelators that we used to prevent the IL-4-dependent OL differentiation bind more stably to zinc ions than to manganese ions (the TPEN stability constants logK are 15.58 for zinc and 10.27 for manganese; the CaEDTA stability constants logK are 16.4 for zinc and 13.8 for manganese) [54,55]. Therefore, it is unlikely that ZIP11 exerts its function downstream of IL-4 as a manganese transporter in oligodendroglia.

Several studies have reported that MS patients exhibited lower zinc levels in their plasma than healthy controls [4,5,6,7]. Furthermore, in the MS brain, decreased zinc levels were observed in MS lesions, paralleling myelin loss [3]. Additionally, higher STAT6 expression was observed in the normal-appearing white matter of MS patients than in healthy controls, and most STAT6 was localized in OL by immunohistochemistry [19,20]. In a subsequent study, oligodendroglial STAT6 expression was found to be even higher in the lesion sites of MS brains [21]. IL-4 and IL-4-secreting cells were also increased in MS patients [56,57,58]. These findings suggest that IL-4 stimulation occurs in MS lesions under conditions of zinc deficiency. Because of zinc shortage, it may fail to stimulate OL differentiation and efficient remyelination.

## 5. Conclusions

**Figure 8:**
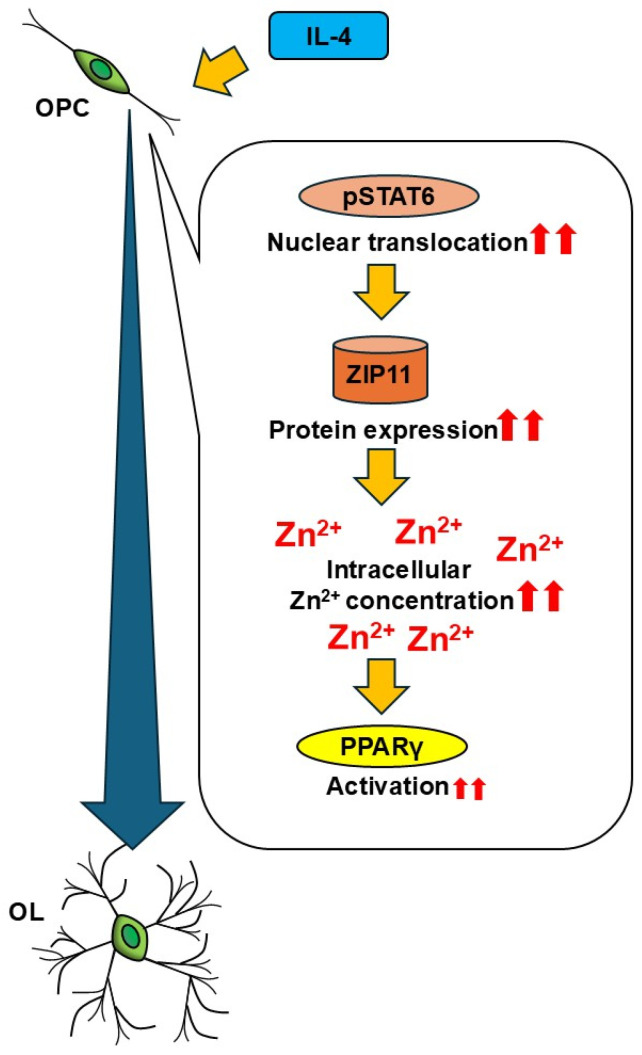
Summary of the study. Exposure to IL-4 activates STAT6 in OPC through phosphorylation and nuclear translocation and leads to ZIP11 upregulation, which in turn induces intracellular zinc concentrations, resulting in PPARγ activation and the subsequent enhancement of OL differentiation. The main stimulatory effects are marked by two red arrows.

In conclusion, our results suggest that the extracellular zinc uptake is essential for IL-4-enhanced OL differentiation, mediated by STAT6 and necessary for PPARγ activation (Figure 8). Furthermore, it is reasonable to assume that zinc signaling is impaired in MS lesion sites. The current study indicates that restitution of zinc signaling in OL could be a potential target for a therapeutic strategy in MS.

## Figures and Tables

**Table 1:** Primer sets for real-time RT-PCR amplification.

Transcript	Primers
*Gapdh*	5′-TCCAGTATGACTCTACCCACG-3′
	5′-CACGACATACTCAGCACCAG-3′
*Stat6*	5′-GCATCTATCAGAGGGACCCC-3′
	5′-GGGAAGTGGGGTAGCACAAT-3′
*Slc39a8*	5′-TGCCCAGCATACTTTGTTCC-3′
	5′-CTTTTGGGTTCCTTGGGAGT-3′
*Slc39a11*	5′-TCGGCTAGCTCTGAGAACCT-3′
	5′-GACCCTGTTACGCTGGTTCA-3′

## Data Availability

All data generated or analyzed during this study are included in this published article.

## Supplementary Materials

The following supporting information can be downloaded at https://www.mdpi.com/article/10.3390/cells14221756/s1, Figure S1. Colocalization of markers in primary oligodendroglial cultures.; Figure S2. Representative images for Figure 7.

## Abbreviations

The following abbreviations are used in this manuscript:

CaEDTA	Calcium ethylenediaminetetraacetate
CNS	Central nervous system
DAPI	4′, 6′-diamidino-2-phenylindole
FGF2	Basic fibroblast growth factor
GAPDH	Glyceraldehyde 3-phosphate dehydrogenase
JAK	Janus kinase
MAG	Myelin-associated glycoprotein
MBP	Myelin basic protein
MS	Multiple sclerosis
OL	Oligodendrocyte
Olig2	Oligodendrocyte transcription factor 2
OPC	Oligodendrocyte progenitor cells
PDGF-AA	Platelet-derived growth factor AA
PLP1	Proteolipid protein 1
PPARγ	Peroxisome proliferator-activated receptor gamma
pSTAT6	Phospho-STAT6
STAT6	Signal transducer and activator of transcription 6
TPEN	*N*,*N*,*N′*,*N′*-tetrakis(2-pyridylmethyl)ethylenediamine
ZIP	Zrt, Irt-like protein
ZnT	Zinc transporter
