# Supplementary material for: Interleukin-4-Enhanced Oligodendrocyte Differentiation Depends on Extracellular Zinc Uptake via ZIP11
Source: Cells. 2025 Nov 10;14(22):1756. doi: 10.3390/cells14221756 (PMC12651824; doi:10.3390/cells14221756)
Supplement: Supplementary file 1 [file cells-14-01756-s001.zip › cells-3845346-supplementary.pdf]

Supplementary Materials

Interleukin-4-enhanced oligodendrocyte differentiation depends on extracellular zinc uptake via ZIP11

Takaaki Aratake, Serika Kurita and Michael Wegner *
Institut für Biochemie, Friedrich-Alexander-Universität Erlangen-Nürnberg, 91054 Erlangen, Germany; takaaki.aratake@fau.de (T.A.); serika.aratake@fau.de (S.K.)
*Correspondence: michael.wegner@fau.de (M.W.); Tel.: +49-9131-8524620 (M.W.)

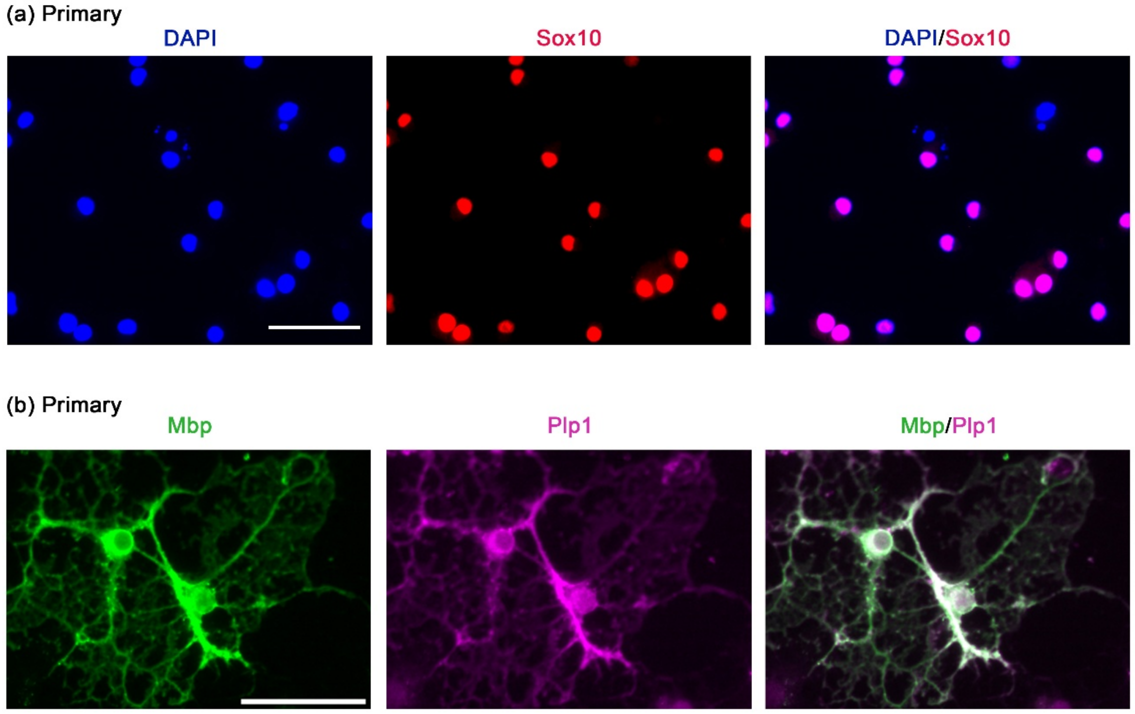

Figure S1. Co-localization of markers in primary oligodendroglial cultures. After 1 day in culture, cells were differentiated for 3 days. Immunocytochemistry was performed for the pan-oligodendroglial marker Sox10 and DAPI (a) and the myelin and mature OL markers Mbp and Plp1 (b). Scale bar = 50 µm.

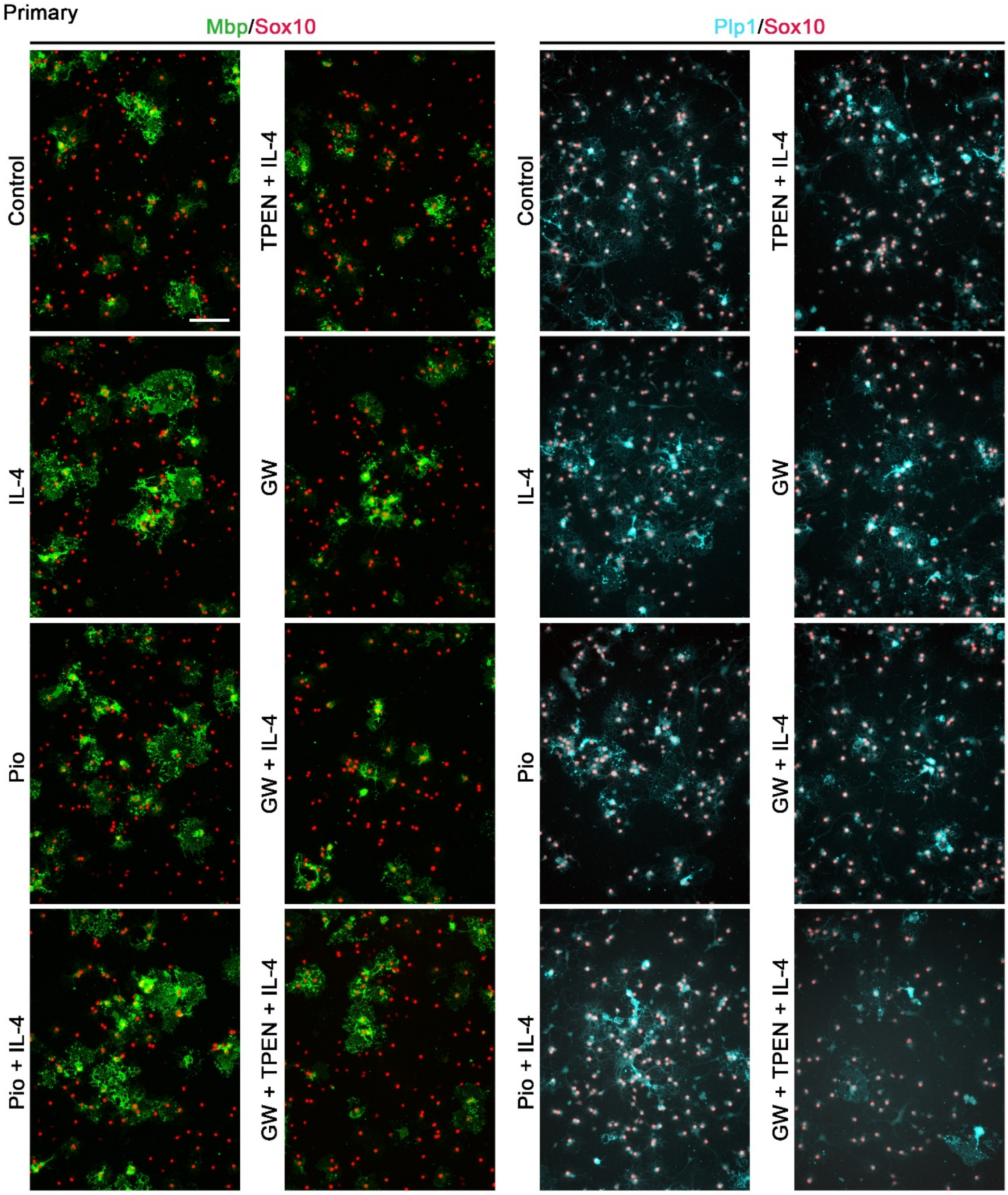

Figure S2. Representative images for Figure 7. After 1 day in culture, primary oligodendroglial cells were switched to differentiation medium in the presence of 1 µM GW9662 (GW), 1 µM pioglitazone (Pio), 1 µM TPEN and 20 ng/mL IL-4 or various combinations thereof for 3 days. Differentiation was monitored by immunochemical visualization of Mbp (green) and Plp1 (blue) in cells with Sox10-positive nuclei (red). Scale bar: 100 µm.
